# ERα-mediated alterations in circ_0023642 and miR-490-5p signaling suppress bladder cancer invasion

**DOI:** 10.1038/s41419-019-1827-3

**Published:** 2019-08-27

**Authors:** Longxiang Wu, Mengda Zhang, Lin Qi, Xiongbing Zu, Yuan Li, Longfei Liu, Minfeng Chen, Yangle Li, Wei He, Xiheng Hu, Miao Mo, Zhenyu Ou, Long Wang

**Affiliations:** Departments of Urology, Xiangya Hospital, Central South University, 410008 Changsha, China

**Keywords:** Bladder cancer, Bladder cancer

## Abstract

Epidemiological studies show obvious gender differences in the incidence and the prognosis of bladder cancer (BCa). Estrogen receptor alpha (ERα) was recently shown to play a protective role in BCa. However, the mechanisms by which ERα mediates BCa progression need to be further elucidated. In the present study, we explored the mechanisms by which ERα inhibits BCa invasion by modulating circRNA levels. ERα suppressed BCa invasion by decreasing circ_0023642 expression. Chromatin immunoprecipitation (ChIP) and luciferase assays revealed that ERα reduced circ_0023642 expression by regulating the expression of its host gene, UVRAG, at the transcriptional level. ERα decreased circ_0023642 levels and subsequently increased miR-490-5p expression, resulting in decreased EGFR expression to suppress BCa cell invasion. Circ_0023642 was demonstrated to directly bind to miR-490-5p. Notably, miR-490-5p regulated EGFR expression by binding to the miR-490-5p-binding site located in the 3′-untranslated region (UTR) of the EGFR mRNA. Preclinical studies using an in vivo mouse model also confirmed that this ERα/circ_0023642/miR-490-5p/EGFR signaling pathway suppressed BCa progression. Altogether, this newly identified pathway may serve as the basis for developing novel therapeutic strategies to treat BCa.

## Introduction

Bladder cancer (BCa) is the tenth most common malignancy worldwide, with over an estimated 549,000 new cases and 200,000 deaths reported in 2018. Epidemiological studies have shown obvious gender differences: the incidence and metastasis of BCa is ~4-fold higher in men than in women^1^. This sex disparity implies the potential involvement of sex steroid pathways in bladder cancer development and progression^2^. Estrogen receptors (ERs) might be related to BCa initiation and progression^3^. The two major types of ERs, ERα and ERβ, were recently shown to play different roles in BCa development. ERα protects against BCa, and ERβ promotes the development of BCa^4,5^. However, the mechanisms by which ERs mediate BCa progression remain to be further elucidated.

Circular RNAs (circRNAs) are a large class of endogenous noncoding RNAs that are generated as covalently closed loop structures with neither 5′ caps nor 3′ tails^6^. Compared to linear RNAs, circRNAs are highly stable and resistant to RNase R digestion. Based on accumulating evidence, circRNAs are important regulators of gene expression and are involved in the occurrence and progression of various cancers^7^. One important mechanism of action of circRNAs is to function as competing endogenous RNAs (ceRNAs) that interact with microRNAs (miRNAs) and regulate the expression of target messenger RNAs (mRNAs)^8^. In addition, circRNAs may bind to RNA-binding proteins or undergo translation to modulate gene expression. The circRNA-miRNA-mRNA axis is a well-studied regulatory mechanism by which circRNAs potentially affect tumor progression^9^.

Recent studies reflect the broad interest of the scientific community in the roles of circRNAs in BCa. Many circRNAs have been shown to be closely related to BCa progression^10,11^. ERα inhibits BCa invasion; however, researchers have not determined whether ERα exerts its function by regulating circRNAs.

In this study, we analyzed the changes in the expression of BCa-related circRNAs after modulating ERα levels in BCa cells. We identified circ_0023642 as a downstream target of ERα that affects BCa invasion by acting as a miR-490-5p sponge and regulating EGFR expression. Here, we report a novel mechanism by which ERα modulates circ_0023642/miR-490-5p signaling to suppress BCa cell invasion.

## Methods

### Cell lines

The human BCa cell lines J82 and UMUC3 were purchased from the American Type Culture Collection (ATCC, Manassas, VA). These cells were cultured in Dulbecco’s Modified Eagle’s Media (DMEM) supplemented with 10% FBS, 2 mM L-glutamine, 100 IU/mL penicillin, and 50 μg/mL streptomycin at 37 °C with a 5% CO_2_ atmosphere.

### Invasion assay

Transwell chambers with 8 μm pore size polycarbonate membranes (Corning Incorporated, Corning NY) were coated with diluted Matrigel (BD Biosciences, Sparks, MD) and used for the invasion assay. A total of 5 × 10^4^ BCa cells were collected in serum-free media and placed in the upper chambers. Seven-hundred-fifty microliters of media containing 10% FBS were added to the lower chambers. After 24 h of incubation at 37 °C in a 5% (v/v) CO_2_ atmosphere, cells that invaded through the transwell chambers were permeabilized with methanol and stained with 0.1% crystal violet. The invading cells were counted from six random microscopic fields. Quantitative results indicate the means ± SD of triplicates.

### RNA extraction and quantitative real-time PCR analysis

Total RNA was extracted from cells using TRIzol reagent (Invitrogen, Grand Island, NY) according to the manufacturer’s instructions. We used 2 µg of RNA for reverse transcription reactions using Superscript III transcriptase (Invitrogen). Quantitative real-time PCR (qRT-PCR) was performed using a Bio-Rad CFX96 system with SYBR green to measure the mRNA expression level of the genes of interest. Expression levels were normalized to the expression of the housekeeping genes GAPDH and U6. For the RNase R treatment, 2 µg of total RNA was incubated for 15 min at 37 °C with or without 3 U/mg RNase R (Epicentre Technologies, Madison, WI, USA).

### Luciferase reporter assay

The 2 kb promoter region of the human UVRAG gene was cloned into a PGL3 firefly luciferase plasmid (Promega, Madison, WI). The QuikChange mutagenesis kit was used for site-directed mutagenesis of the ERα-binding site in the 5′-region of the UVRAG promoter. J82 and UMUC3 cells were plated in 24-well plates, and cDNAs or short-interfering RNAs were transfected using the Lipofectamine 3000 transfection reagent (Invitrogen, Carlsbad, CA) according to the manufacturer’s instructions. Fragments of the EGFR 3′-UTR containing wild-type or mutant miRNA-responsive elements were cloned into a PsiCheck2 vector (Promega) downstream of the Renilla luciferase ORF. A luciferase reporter vector containing miR-490-5p mimics or miR-490-5p inhibitors was transfected into J82 or UMUC3 cells. The thymidine kinase promoter-Renilla luciferase reporter plasmid (pRL-TK) was used as the internal control. Luciferase activity was measured 48 h after transfection using a Dual-Luciferase Assay kit (Promega) according to the manufacturer’s instructions.

### Chromatin immunoprecipitation assay (ChIP)

Cells were crosslinked with 1% formaldehyde. Ten minutes later, 1.375 M glycine was added to the culture medium to stop the reaction. Samples were sonicated to shear the DNA into 300–1000 bp fragments. Products were preincubated with Protein A-agarose conjugated with normal rabbit IgG. An anti-ERα antibody was used for immunoprecipitation. IgG was used as a negative control. Specific primers were designed to amplify target sequences within the human UVRAG promoter. PCR products were analyzed using agarose gel electrophoresis.

### Biotin-coupled probe pull-down assay

A biotin-labeled probe was designed to bind to the circular junction of circ_0023642. An oligo probe was used as a control. Cells were collected and lysed in lysis buffer. Then, the cell lysate was rotated overnight at 4 °C after adding 1.5 mL of RNase inhibitor and 500 pM biotinylated probes. Cell lysates were then incubated with streptavidin-coated magnetic beads (Life Technologies, USA) for 2 h to pull down the biotin-coupled RNA complexes. The beads were washed with lysis buffer five times, and the total RNA bound to the beads was extracted with TRIzol and then subjected to a qRT-PCR analysis to examine the circ_0023642 miRNAs that were pulled down.

### In vivo studies

J82 cells were stably transfected with a luciferase reporter gene (pcDNA3.0-luciferase). Thirty-two 6- to 8-week-old female mice were randomly divided into four groups. Orthotopic xenografts of these J82 cells were generated as described in a previous study. A total of 1 × 10^6^ J82 cells from different groups were injected into the bladder wall muscle after resuspension in phosphate-buffered saline (PBS) and mixing with Matrigel (1:1). Tumor growth and metastasis were monitored weekly with a fluorescent Imager (IVIS Spectrum, Caliper Life Sciences, Hopkinton, MA). Eight weeks later, the mice were sacrificed and the primary tumors and metastases were further examined. All procedures using animals were performed in strict accordance with the guidelines of the Animal Center of Central South University, and all animal experimental procedures were approved by the Experimental Animal Ethical Committee of Central South University.

### Statistical analysis

The data are presented as the means ± SD from at least three independent experiments. All analyses were performed with SPSS 17.0 software (SPSS Inc., Chicago, IL). Differences in data between two groups were analyzed using unpaired Student’s *t-*tests. *P* < 0.05 was considered statistically significant.

## Results

### ERα decreases bladder cancer cell invasion

**Fig. 1:**
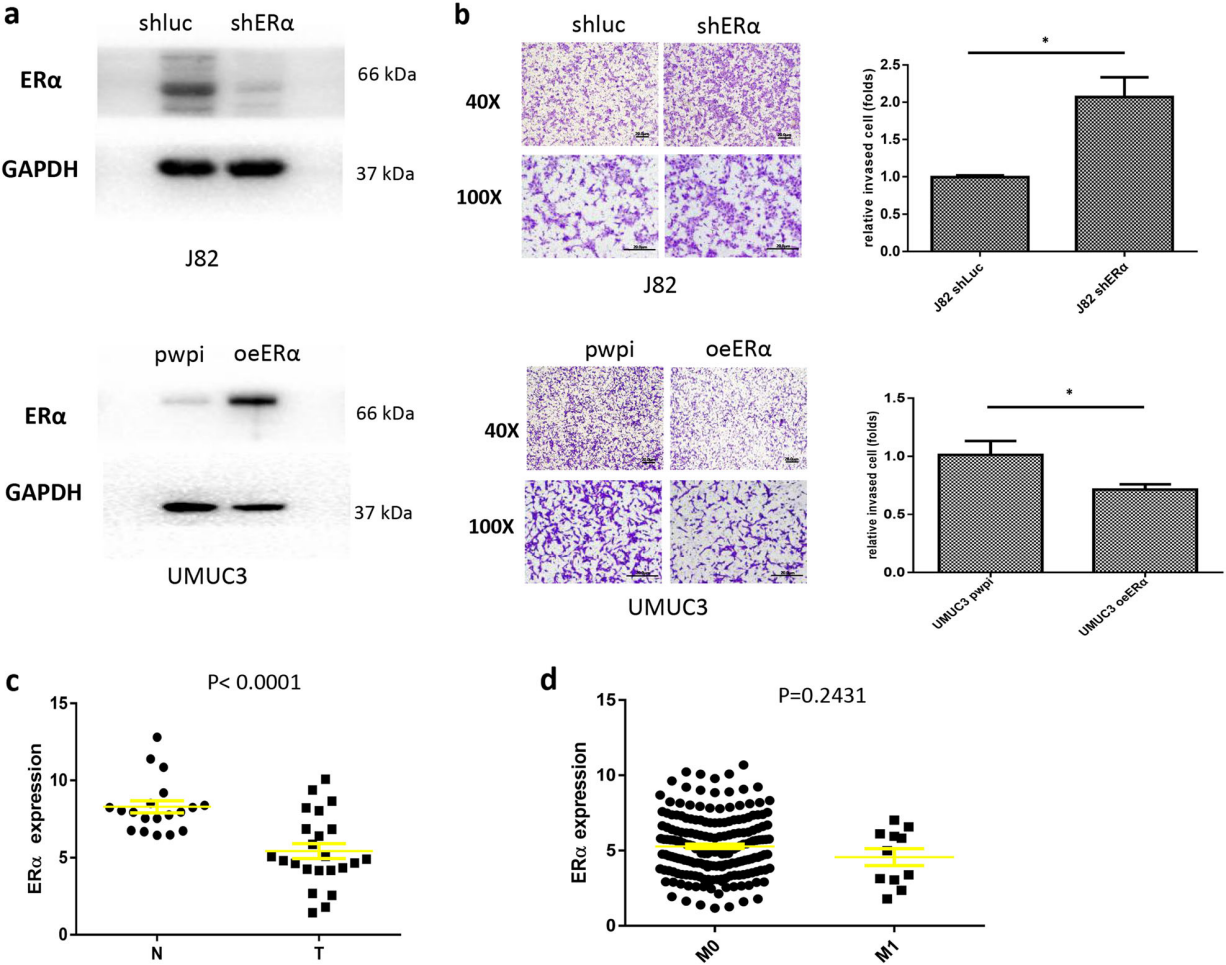
ERα decreases BCa invasion. **a** J82 cells were transduced with lentivirus carrying shERα to knock down ERα expression or shLuc as a control. UMUC3 cells were transduced with a lentivirus designed to overexpress ERα (oeERα) or with a vector control (Ctrl) and used in subsequent experiments. Western blot analyses were performed, and proteins were detected with an antibody against ERα. **b** Matrigel invasion assays were performed using J82 and UMUC3 cells transduced with the lentiviruses described above. **c** We used the TCGA database to analyze ERα expression in BCa samples, and the results revealed higher ERα expression in BCa tumors than in normal bladder tissues. **d** An analysis of the TCGA database revealed the expression of the ERα mRNA in patients with BCa presenting metastatic foci or patients without metastasis. Data shown in **b** and **c** are presented as means ± SD. *P < 0.05

According to previous studies, ERα functions as a tumor suppressor during bladder cancer progression. We knocked down ERα expression in J82 cells and overexpressed ERα in UMUC3 cells to assess the effects of ERα on BCa cell and invasion. A western blot assay was used to verify the levels of the ERα protein in the different groups of BCa cells (Fig. 1a). We used a matrigel-coated transwell invasion assay to examine the effects of ERα on BCa invasion. ERα knockdown in J82 cells increased cell invasion, while ERα overexpression in UMUC3 cells decreased cell invasion (Fig. 1b), compared to the controls.

Taken together, the results from these experiments using various BCa cells reveal that ERα decreases BCa cell invasion.

### Human clinical data imply that lower ERα expression is associated with a worse prognosis in patients with BCa

Human clinical data from The Cancer Genome Atlas (TCGA) database (http://cancergenome.nih.gov) showed lower ERα expression in bladder tumor tissues than in normal tissues (*P* < 0.0001, Fig. 1c). Our analysis also showed lower expression of the ERα mRNA in patients with BCa presenting metastatic foci than in patients without metastasis, although the differences were not significant (*P* = 0.2431, Fig. 1d).

In summary, the results from the analysis of the TCGA human clinical data suggest that lower ERα expression is associated with disease progression in patients with BCa, consistent with our in vitro data from multiple types of BCa cells showing that ERα decreases BCa cell invasion.

### ERα decreases BCa cell invasion by downregulating circ_0023642

**Fig. 2:**
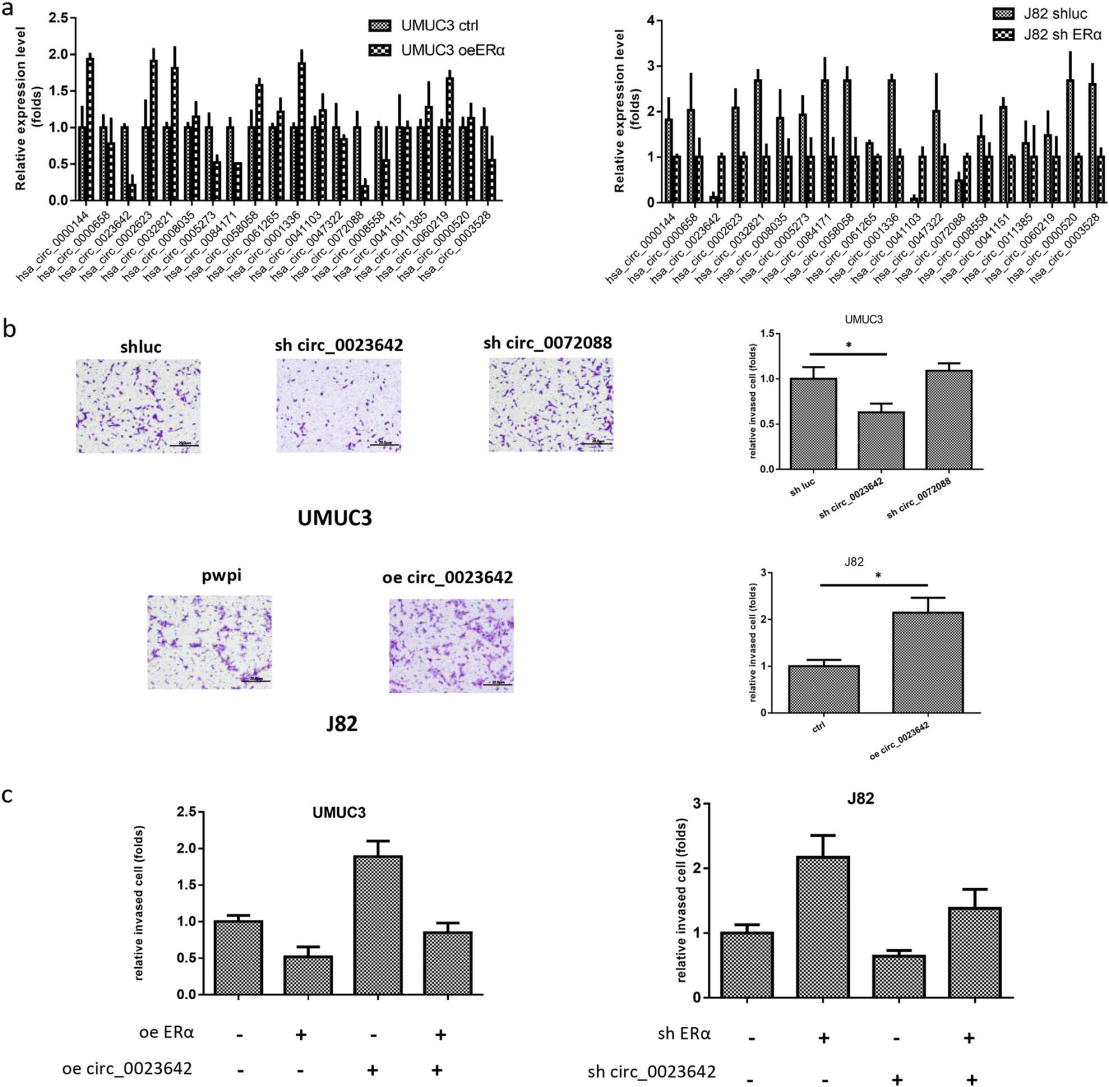
ERα decreases BCa cell invasion by downregulating circ_0023642. **a** Real-time PCR analysis of circRNAs related to BCa progression in UMUC3 cells overexpressing ERα or the vector (Ctrl) and J82 cells expressing shERα or the shLuc control (Ctrl). **b** Among the circRNAs screened, circ_0023642 and circ_0072088 expression were downregulated to the greatest extent by ERα. We knocked down circ_0023642 and circ_0072088 in UMUC3 cells and overexpressed circ_0023642 in J82 cells. Matrigel invasion assays were performed using UMUC3 and J82 cells that were transduced with the vectors described above. **c** Matrigel invasion assays were performed using UMUC3 cells transfected with/without oeERα or circ_0023642 and J82 cells transfected with/without shERα or shcirc_0023642. Values are presented as relative invasion (fold). *P < 0.05

We focused on circRNAs to dissect the molecular mechanism by which ERα decreases BCa invasion, since a growing body of evidence indicates that circRNAs have critical functions in tumor progression. A previous study identified the differentially expressed circRNAs in BCa using a microarray assay. We selected the top 20 circRNAs ranked by fold change from the microarray and investigated, which circRNAs were regulated by ERα using qRT-PCR. Notably, the expression of circ_0023642 and circ_0072088 was significantly decreased when ERα was overexpressed in UMUC3 cells but was significantly increased when ERα was knocked down in J82 cells (Fig. 2a) compared to that in the controls.

We then investigated the effects of circ_0023642 and circ_0072088 on BCa cell invasion. We constructed a circ_0023642-targeted short-hairpin RNA (shRNA) and circ_0072088-targeted shRNA to knock down these two circRNAs in BCa cells. As shown in Fig. 2b, knockdown of circ_0023642, but not circ_0072088, significantly decreased UMUC3 cell invasion compared to that in the controls. Furthermore, overexpression of circ_0023642 increased J82 invasion (Fig. 2b) compared to that in the controls.

We then performed rescue experiments to examine the effects of circ_0023642 on ERα-mediated BCa cell invasion. ERα knockdown in J82 cells increased J82 cell invasion; furthermore, circ_0023642 knockdown partially reversed the ERα knockdown-induced increase in BCa cell invasion. Using an opposite approach, ERα overexpression in UMUC3 cells decreased invasion, and overexpression of circ_0023642 partially reversed the oeERα-induced decrease in the invasion of UMUC3 cells (Fig. 2c).

Altogether, the results obtained from two BCa cell lines using different approaches suggest that ERα may decrease BCa cell invasion by altering circ_0023642 expression.

### Dissection of the mechanism by which ERα regulates circ_0023642 expression

CircRNAs are derived from pre-mRNAs that are transcribed from their host genes. The expression of a circRNA is related to the expression of its host gene^12^. We then studied the potential mechanism by which ERα regulates the expression of the circ_0023642 host gene, UVRAG.

**Fig. 3:**
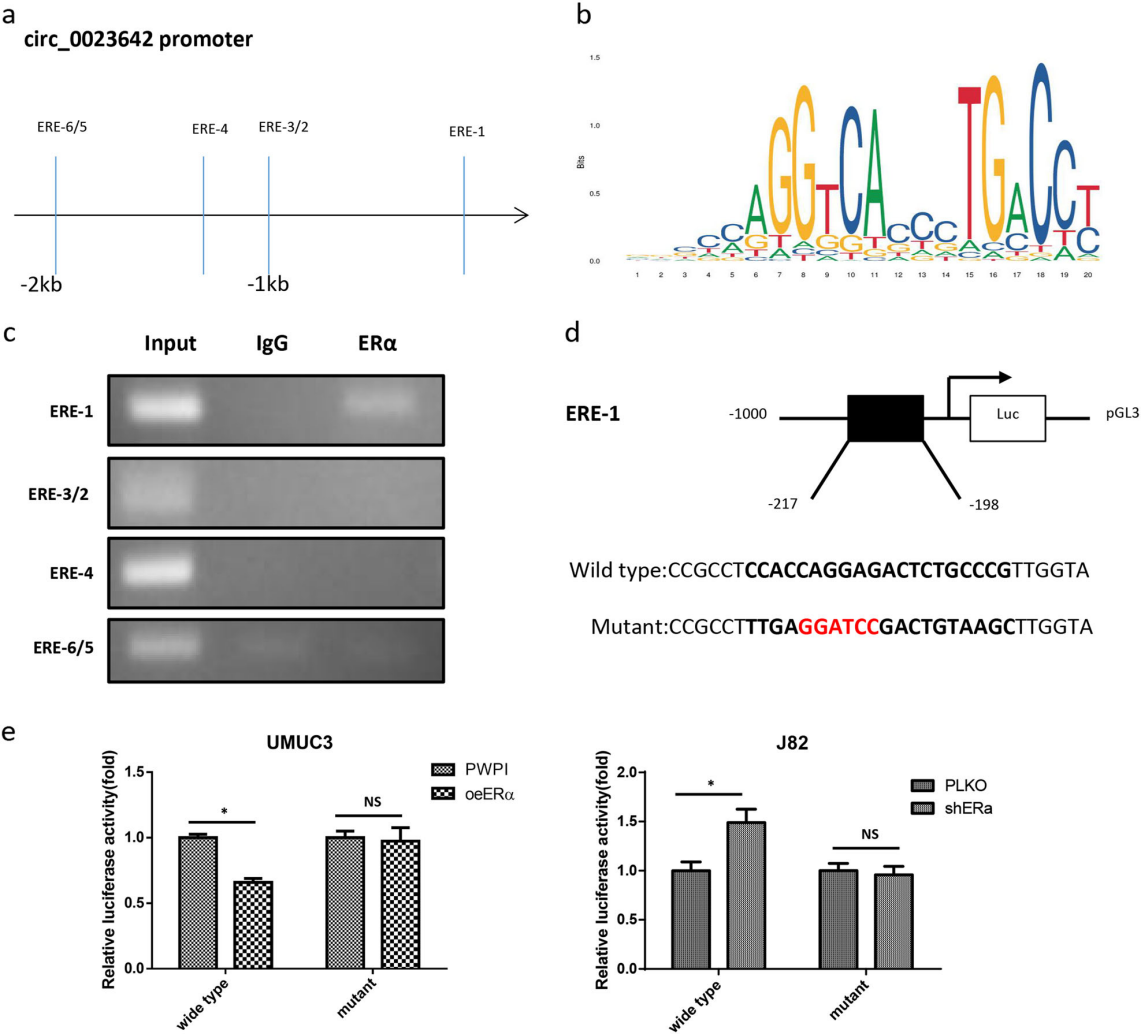
Determination of the mechanism by which ERα regulates circ_0023642 expression. **a** Schematic depicting the potential ERα-binding sites in the 5′-region of the UVRAG promoter, the host gene of circ_0023642. **b** The potential estrogen response elements (EREs) in the promoter region of UVRAG predicted by JASPAR. **c** The ChIP assay revealed that ERα bound to the 1st potential ERE-binding site in the UVRAG promoter. IgG antibody pull-down was used as a negative control. **d** Schematic showing the procedure used to clone the 1 kb UVRAG promoter (wild-type or mutant) into the pGL3 basic luciferase reporter vector (pGL3). **e** Cotransfection of wild-type or mutant ERE UVRAG promoter pGL3-Luciferase constructs into UMUC3 cells with/without oeERα and into J82 cells with/without shERα. The luciferase assay was used to detect promoter activity. *P < 0.05, NS-not significant compared to the controls

We used the Ensembl and JASPAR (http://jaspar.genereg.net/) web-based approaches to predict the potential estrogen response elements (EREs) in the 2 kb 5′-region of the UVRAG promoter. The bioinformatics analysis identified six potential EREs in the 2 kb UVRAG promoter region (Fig. 3a, b). A ChIP assay was used to analyze the binding of ERα to these predicted EREs. ERα specifically bound to ERE1 (Fig. 3c). We then cloned a 2 kb region of the promoter into the pGL3 vector and mutated the ERE1 sequences for a luciferase reporter assay (Fig. 3d). Overexpression of ERα decreased luciferase reporter activity in UMUC3 cells with the wild-type ERE1 sequence, but not in UMUC3 cells with the mutant ERE1 sequence. Furthermore, knockdown of ERα in J82 cells increased wild-type ERE luciferase reporter activity, but not mutant ERE luciferase reporter activity (Fig. 3e).

Based on these results, ERα modulates circ_0023642 expression by modulating the expression of its host gene, UVRAG, after binding to the ERE in the 5′-region of the UVRAG promoter.

### ERα inhibits BCa cell invasion by altering circ_0023642/miR-490-5p signaling

One prominent mechanism by which circRNAs function is to sponge miRNAs and sequester them from protein-coding mRNAs. Subsequently, we focused on miRNAs to determine the mechanism by which ERα-modulated circ_0023642 expression decreased BCa cell invasion.

We used publicly available bioinformatics prediction databases (http://circinteractome.nia.nih.gov/) and identified several miRNAs (miR-223-3p, miR-490-5p, miR-508-3p, miR-576-3p, miR-616-3p, and miR-653-5p) that may interact with circ_0023642 in BCa.

**Fig. 4:**
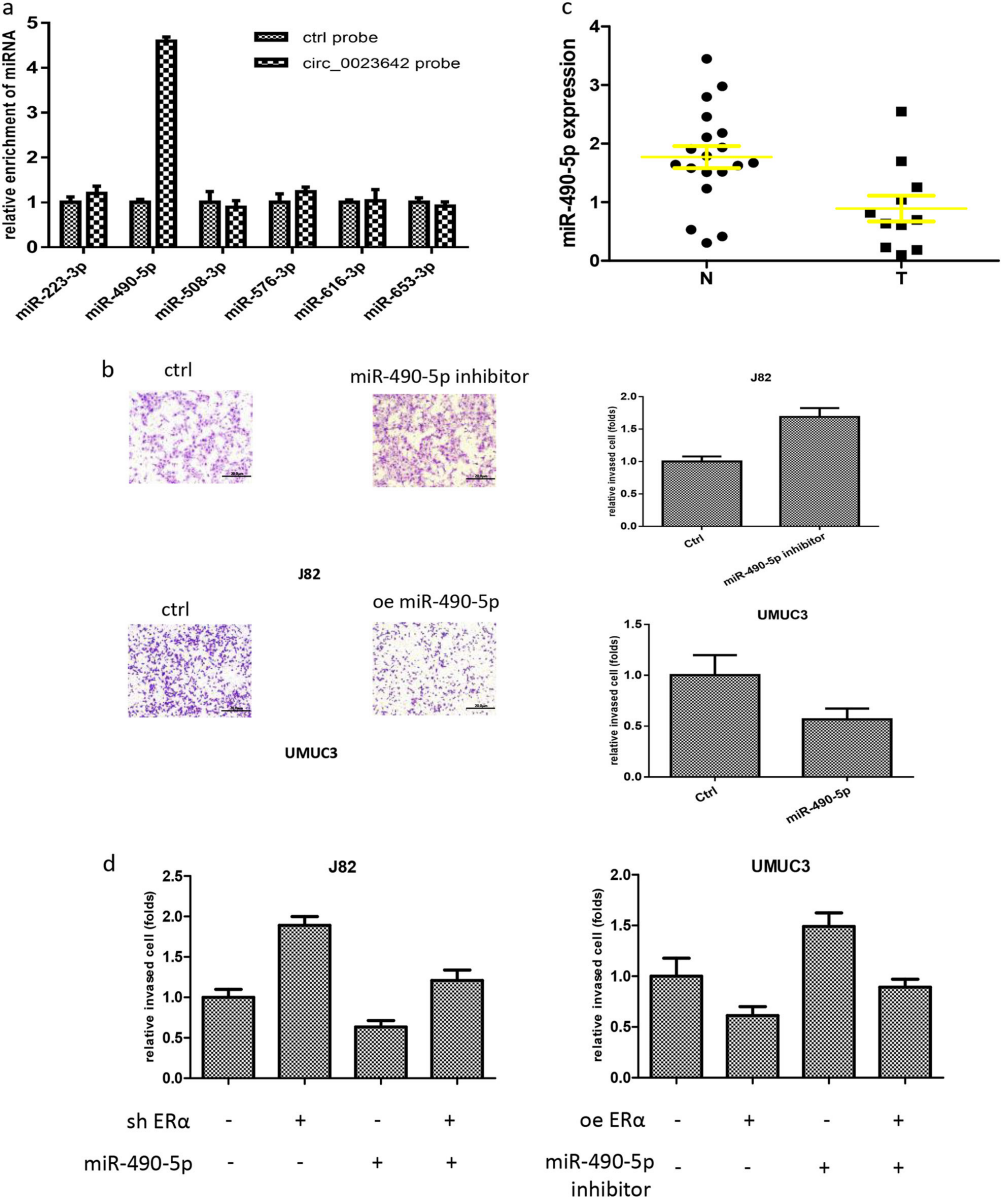
ERα inhibits BCa cell invasion by altering circ_0023642/miR-490-5p signaling. **a** RNA pull-down assays were performed with biotinylated oligonucleotides designed to target the circ_0023642 circular junction; miR-490-5p was enriched in the pull-down products. **b** We transfected a miR-490-5p inhibitor into J82 cells and a miR-490-5p mimic into UMUC3 cells. Matrigel invasion assays were performed in J82 and UMUC3 cells transfected with the aforementioned constructs. **c** An analysis of the TCGA database revealed miR-490-5p expression in BCa tumor tissues compared to normal bladder tissues. **d** Ectopic miR-490-5p expression reversed the shERα-induced decrease in the invasion of J82 cells. Transfection of UMUC3 cells with a miR-490-5p inhibitor reduced the ERα-induced decrease in cell invasion. Data are presented as means ± SD

We then used an RNA pull-down assay to test whether circ_0023642 interacted with those candidate miRNAs. Biotinylated oligonucleotides (5′) were designed to target the circ_0023642 circular junction. Only miR-490-5p was enriched in the pull-down products and directly interacted with circ_0023642 (Fig. 4a).

We examined the effects of miR-490-5p on BCa cell invasion. Overexpression of miR-490-5p decreased UMUC3 invasion, while inhibition of miR-490-5p increased J82 invasion, compared to that in the controls (Fig. 4b). Importantly, the results from TCGA databases further revealed that BCa expressed miR-490-5p at lower levels than normal bladder tissues (Fig. 4c), suggesting that miR-490-5p might suppress BCa progression. We further examined the effects of miR-490-5p on ERα-mediated BCa cell invasion. We modulated the expression of ERα and miR-490-5p in BCa cells and conducted a transwell invasion assay. Overexpression of miR-490-5p partially reversed the shERα-induced increase in the invasion of J82 cells; in contrast, a miR-490-5p inhibitor blocked the effects of overexpression of ERα and decreased BCa invasion in UMUC3 cells (Fig. 4d).

Thus, the ERα/circ_0023642 axis may function by modulating miR-490-5p expression to alter BCa cell invasion.

### ERα/circ_0023642/miR-490-5p signaling decreases BCa cell invasion by altering EGFR signaling

**Fig. 5:**
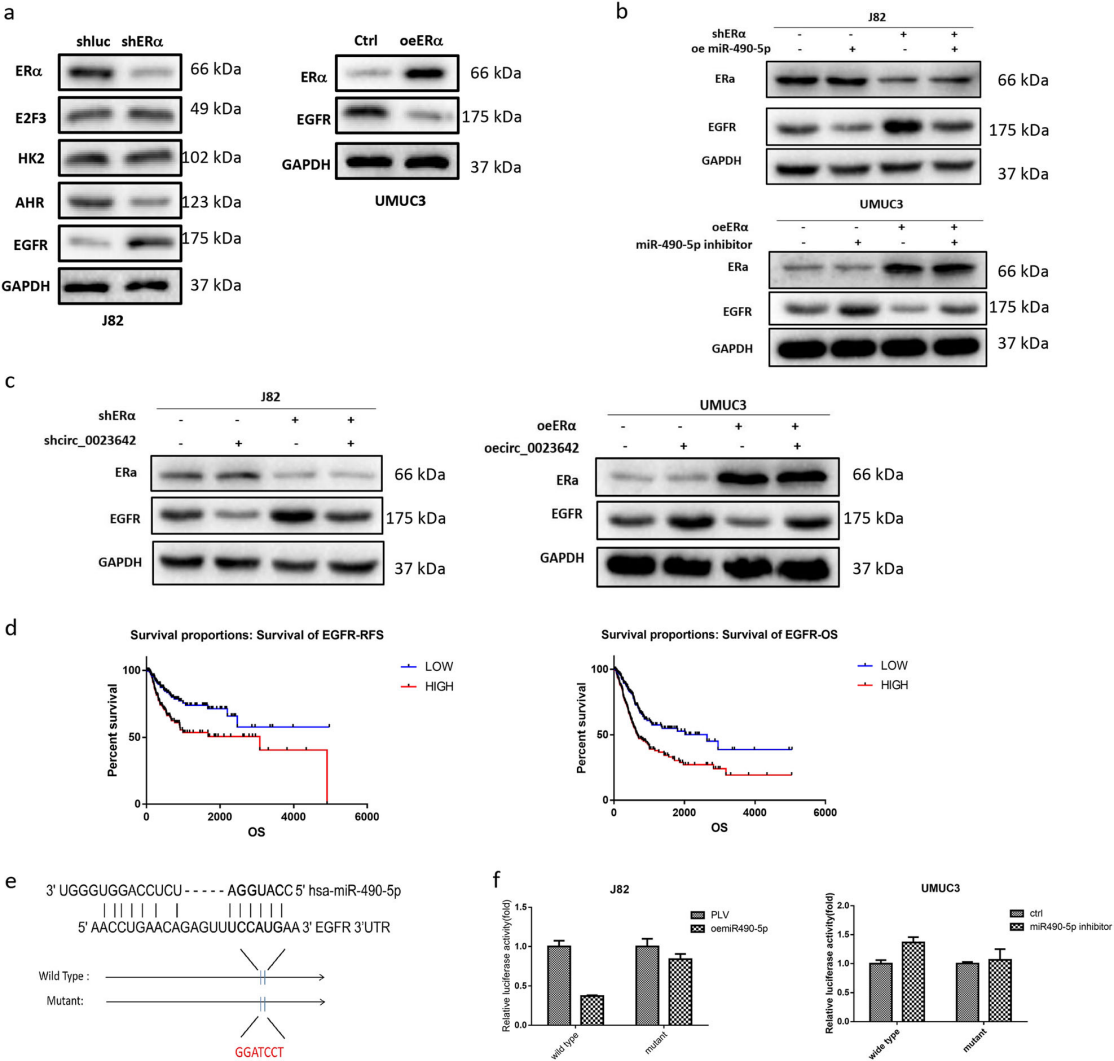
ERα/circ_0023642/miR-490-5p signaling decreases BCa cell invasion by altering EGFR signaling. **a** Western blots showing the levels of four metastasis-related proteins in J82 cells transfected with shERα compared with shLuc (left panel) and in A498 cells transfected with oeERβ compared with pWPI (right panel), and levels of the EGFR protein in UMUC3 cells overexpressing ERα or the vector (Ctrl). **b** Western blots showing ERα and EGFR levels after the modulation of ERα and miR-490-5p expression in J82 and UMUC3 cells. **c** Western blots showing ERα and EGFR levels after the modulation of ERα and circ_0023642 expression in J82 and UMUC3 cells. **d** An analysis of the TCGA database revealed differences in overall survival and relapse-free survival between patients with higher EGFR mRNA expression and patients with lower EGFR mRNA expression. **e** EGFR 3′-UTRs containing wild-type or mutant miRNA-binding sites were cloned into the psiCheck2 vector. **f** We cotransfected miR-490-5p/miR-490-5p inhibitor and psiCheck2–EGFR 3′-UTR constructs containing WT or mutant miR-490-5p-binding sites into J82 cells/UMUC3 cells, and the luciferase activity was assayed

We searched for metastasis-related genes linked to ERα, circ_0023642, or miR-490-5p in several databases (circBase, CircNet, and OncoLnc) to further investigate the mechanism by which the ERα/circ_0023642/miR-490-5p axis decreases BCa cell invasion. We focused on four metastasis-related genes, E2F3, HK2, AHR, and EGFR, and examined their expression using western blotting. EGFR levels exhibited the greatest change in J82 cells transfected with shERα, which exhibited increased expression of EGFR. On the other hand, EGFR expression was significantly decreased in UMUC3 cells overexpressing ERα (Fig. 5a).

Importantly, the ERα-shRNA-induced increase in EGFR expression was reversed by the addition of miR-490-5p to J82 cells. The decrease in EGFR expression induced by ERα overexpression was also partially reversed in UMUC3 cells transfected with a miR-490-5p inhibitor (Fig. 5b). Furthermore, circ_0023642 knockdown increased the levels of the EGFR protein and partially reversed the ERα-shRNA-induced increase in EGFR expression in J82 cells. In contrast, in UMUC3 cells, circ_0023642 overexpression increased the levels of the EGFR protein and reversed the ERα-induced decrease in EGFR expression (Fig. 5c). An analysis of human clinical data from the TCGA database revealed that patients presenting higher expression of the EGFR mRNA had significantly decreased overall survival and relapse-free survival rates (Fig. 5d).

We identified a potential binding site in the 3′-UTR of the EGFR mRNA to further study the molecular mechanism by which miR-490-5p modulates the level of the EGFR protein (Fig. 5e). We then performed the luciferase reporter assay with the psiCHECK2 vector carrying either the wild-type (WT) or mutant miRNA-target sites. Importantly, miR-490-5p decreased the luciferase activity of the psiCheck2–EGFR-3′-UTR-WT but had little effect on the activity of the psiCheck2–EGFR-3′-UTR-mutant (Fig. 5f). Based on the results of the luciferase reporter assay, miR-490-5p suppresses the expression of the EGFR protein by directly targeting the 3′-UTR of the EGFR mRNA.

### Preclinical studies using an in vivo mouse model reveal that ERα decreases BCa metastasis by altering circ_0023642 expression

**Fig. 6:**
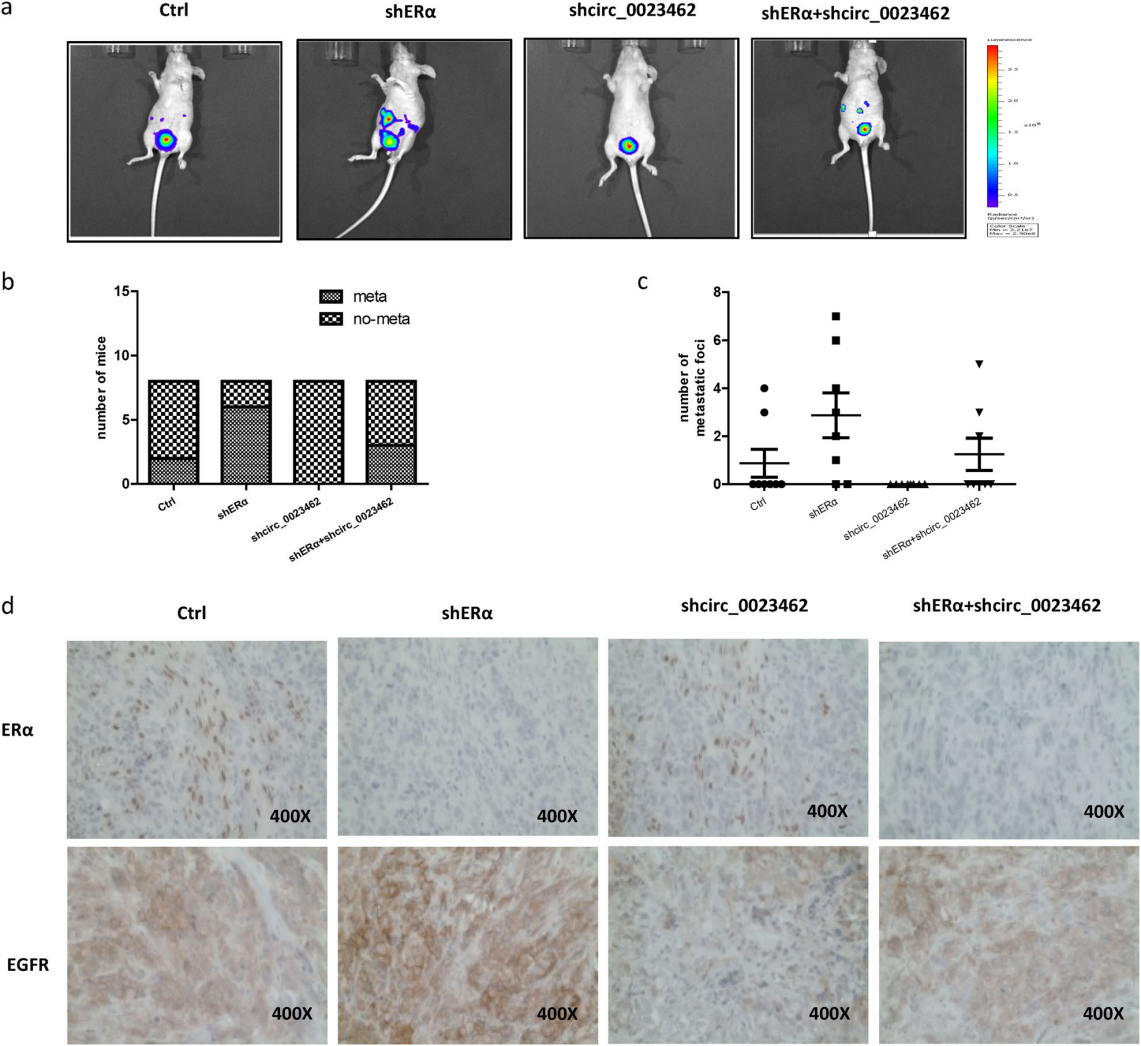
ERα decreases BCa metastasis by altering circ_0023642 expression in vivo. **a** IVIS imaging was used to detect tumor growth and metastasis. Representative IVIS images of the four groups of mice show the outcomes observed at 8 weeks after orthotopic implantation of BCa cells into the bladder wall of female nude mice. **b** Incidence of metastases in different groups of mice at 8 weeks after BCa cell implantation. **c** Comparison of the number of metastatic foci in the different mouse groups. **d** Representative images of IHC staining for ERα and EGFR in orthotopic bladder tumor tissues from the four groups

We used an orthotopic xenograft animal model to further confirm the results from the in vitro experiments. Thirty-two female nude mice were randomly assigned to four groups (8 mice/group). J82 cells expressing firefly luciferase were transfected with a control vector, shERα, circ_0023642, or shERα + circ_0023642 and injected into the bladder wall of nude mice. Tumor growth and metastasis were monitored using a real-time in vivo imaging system (IVIS) (Fig. 6a). Eight weeks after tumor implantation, IVIS imaging showed the highest metastasis incidence in mice implanted with J82-Luc cells expressing shERα. In contrast, the J82-luc-shcirc_0023642 tumor group had the lowest metastasis incidence; shcirc_0023642 reversed the shERα-induced increase in the formation of metastatic foci (Fig. 6b). Then, we sacrificed the mice and carefully counted the metastatic foci. The results of the statistical analysis are shown in Fig. 6c.

The results from IHC staining shown in Fig. 6d also indicated that ERα knockdown increased EGFR expression in bladder tumors, which was partially reversed by shcirc_0023642.

Altogether, the in vivo data indicated that ERα decreases BCa metastasis by modulating circ_0023642/EGFR signaling.

## Discussion

According to epidemiology, BCa clearly displays a gender disparity in terms of incidence and mortality. The incidence and mortality rates are 9.6 and 3.2 per 100,000 in men and 2.4 and 0.9 per 100,000 in women, respectively^1^. Sex steroids and hormone receptors may partially account for the differences in outcomes between men and women with BCa. Two major types of ERs, ERα and ERβ, have been studied in BCa. Although inconsistencies exist, the clinical data mainly show lower expression of ERα in high-grade BCa and BCa invading the muscularis propria than in low-grade BCa^13^. Recently, a meta-analysis including 2049 patients from 13 retrospective studies reported the downregulation of ERα expression in bladder tumors compared with that in nonneoplastic urothelial tissues; ERβ expression was upregulated in high-grade and/or muscle-invasive bladder cancers^14–16^. The majority of studies suggest that ERα suppresses BCa initiation and invasion, whereas ERβ promotes BCa initiation and progression^3,17,18^. Our study used in vitro cell lines and mouse models to further confirm that ERα significantly decreases BCa invasion.

Regarding the involvement of ERα in BCa initiation and progression, this receptor has been proposed to exert its effect on BCa cells through multiple pathways. In the present study, we focused on circRNAs, which play important, newly recognized roles in BCa progression^19,20^.

As a recently discovered subclass of endogenous noncoding RNAs, circRNAs are not simply the junk-products of pre-mRNA splicing but participate in the initiation and development of various diseases, including tumors^21–23^. Dysregulation of circRNAs has been detected in BCa, indicating a vital function for these molecules in BCa progression^10^. Here, we screened 20 circRNAs related to BCa progression and found that the expression of some circRNAs was regulated by ERα. Interestingly, circ_0023642, whose host gene is UVRAG, is a critical downstream factor of ERα. Zhou et al.^24^ reported the upregulation of circRNA_0023642 in gastric cancer tissues and its role in inducing metastasis by promoting the EMT. However, the role of circRNA_0023642 in the progression of BCa has not been made clear. In the present study, overexpression of circRNA_0023642 significantly increased BCa cell invasion. We provide the first evidence that circRNA_0023642 expression is regulated by ERα and this circRNA functions as an oncogene in BCa. Importantly, inhibition of circ_0023642 partially blocks the function of ERα in regulating BCa progression.

CircRNAs may function in different ways to regulate gene expression. A well-characterized function of circRNAs is to inhibit miRNAs by sponging miRNAs^22^. Here, we screened several predicted miRNAs using bioinformatics analysis and confirmed that miR-490-5p bound circ_0023642 by performing a biotin-coupled probe pull-down assay and biotin-coupled miRNA capture. Further functional studies also verified that ERα inhibited BCa invasion partially by modulating circ_0023642/miR-490-5p signaling.

Notably, miR-490-5p has been reported to inhibit the progression of different cancers, including hepatocellular carcinoma, renal cell carcinoma and bladder cancer^25,26^. According to Lan et al.^27^, miR-490-5p functions as a tumor suppressor in BCa by targeting c-FOS. We used a bioinformatics prediction database to identify the target genes that mediate the effects of miR-490-5p and identified EGFR, which might be very important in the development of BCa. EGFR is a member of the tyrosine kinase receptor family and is overexpressed in many epithelial tumors, including non-small cell lung, colorectal, gastric, liver, ovarian, and bladder cancers^28,29^. EGFR expression positively correlates with a higher tumor stage, tumor progression, and poor clinical outcomes in patients with BCa^30^. In the present study, we confirmed that miR-490-5p directly targeted the 3′-UTR of the EGFR mRNA to downregulate EGFR expression and subsequently suppress BCa cell invasion.

## Conclusions

In the current study, the novel circ_0023642/miR-490-5p/EGFR signaling pathway, which is downstream of ERα, was required for the ERα-mediated suppression of BCa progression. This information provides a better understanding of the significance of ERα and the mechanism by which it suppresses BCa progression, which might suggest new strategies for the treatment of BCa.
